# Unusual presentation of primary cutaneous mucinosis with Blaschkoid Hemibody distribution: case report

**DOI:** 10.1093/omcr/omaf244

**Published:** 2025-11-26

**Authors:** Maryam Ghaleb, Kaoutar Benchekroun, Fatima Zahra El Ali, Salim Gallouj, Ouiame El Jouari

**Affiliations:** Dermatology Department, Mohammed VI University Hospital, Faculty of medicine and pharmacy, Abdelmalek Essaâdi University, Route de Rabat, Tangier 90060, Morocco; Dermatology Department, Mohammed VI University Hospital, Faculty of medicine and pharmacy, Abdelmalek Essaâdi University, Route de Rabat, Tangier 90060, Morocco; Dermatology Department, Mohammed VI University Hospital, Faculty of medicine and pharmacy, Abdelmalek Essaâdi University, Route de Rabat, Tangier 90060, Morocco; Dermatology Department, Mohammed VI University Hospital, Faculty of medicine and pharmacy, Abdelmalek Essaâdi University, Route de Rabat, Tangier 90060, Morocco; Dermatology Department, Mohammed VI University Hospital, Faculty of medicine and pharmacy, Abdelmalek Essaâdi University, Route de Rabat, Tangier 90060, Morocco

**Keywords:** Blaschko’s lines, case report, genetic mosaicism, localized mucinosis, primary cutaneous mucinosis

## Abstract

Primary cutaneous mucinosis (PCM) is a rare condition characterized by dermal mucin deposition without systemic disease, thyroid dysfunction, or paraproteinemia. The following report details the case of a 25-year-old female patient who exhibited the presence of firm, flesh-colored nodules and plaques that were strictly confined to the right hemibody, distributed linearly along Blaschko's lines. Laboratory tests revealed no significant findings, and histological analysis confirmed the presence of abundant dermal mucin, as indicated by Alcian Blue staining within a normal epidermis. This strictly unilateral Blachkoid distribution is exceptionally rare in mucinoses and raises the hypothesis of genetic mosaicism as a possible pathogenic mechanism. In contrast to generalized scleromyxedema, which carries systemic risk, this case exemplifies a localized, benign variant. This atypical presentation broadens the clinical spectrum of cutaneous mucinoses and underscores the significance of considering this diagnosis in atypical linear dermatoses.

## Introduction & Objectives

Cutaneous mucinoses are a heterogeneous group of disorders defined by abnormal mucin deposition within the skin. They are traditionally divided into localized forms—also known as lichen myxedematosus—and generalized forms, referred to as scleromyxedema, the latter often associated with systemic involvement and poor prognosis [1]. Localized forms are typically indolent and present as papules, nodules, or plaques, often without extracutaneous manifestations.

Primary cutaneous mucinosis is considered idiopathic when not associated with thyroid disease, connective tissue disorders, or malignancy. While localized variants are well-documented, atypical patterns such as unilateral or blaschkoid distributions are exceedingly rare.

Herein, we report a remarkable case of primary cutaneous mucinosis strictly limited to one hemibody, distributed along Blaschko’s lines, raising questions about genetic mosaicism as a pathogenic mechanism.

## Case report

**Figure 1:**
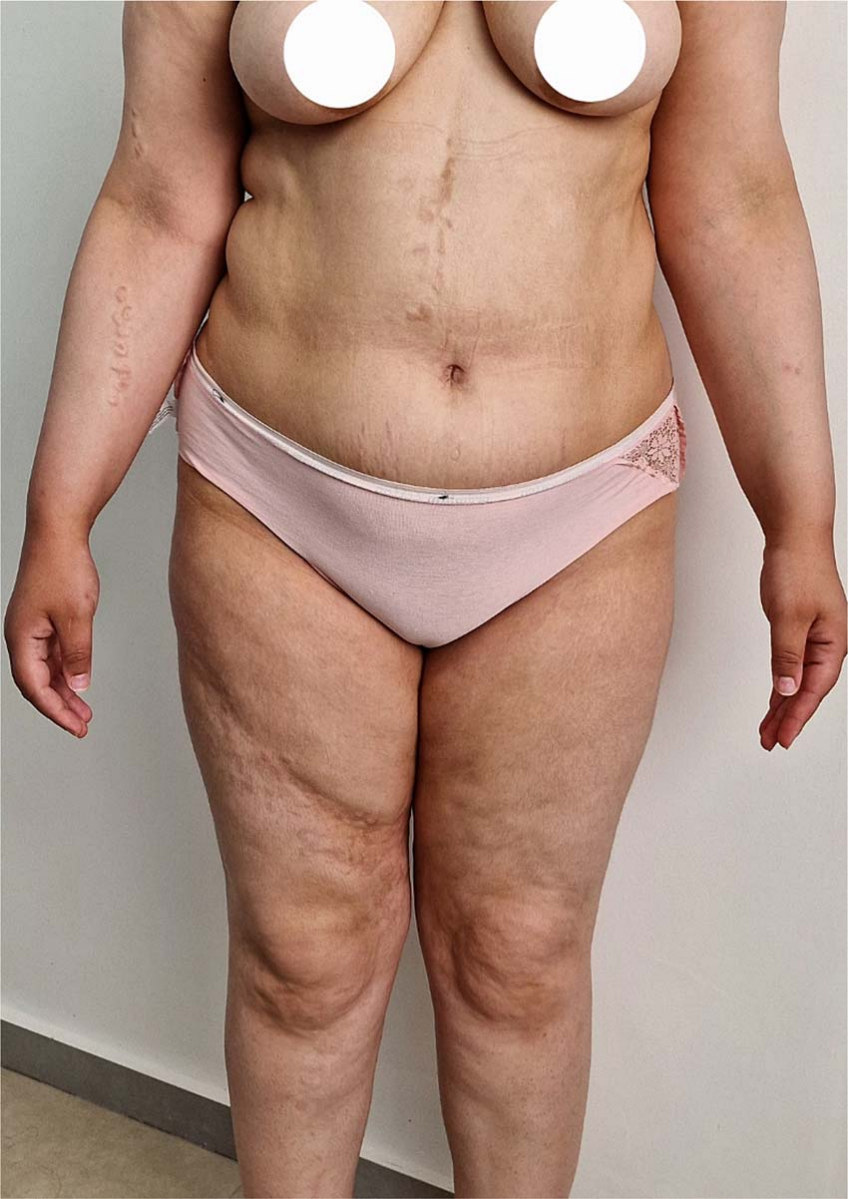
Multiple flesh-colored papulonodules strictly confined to the right hemibody, following Blaschko’s lines.

**Figure 2:**
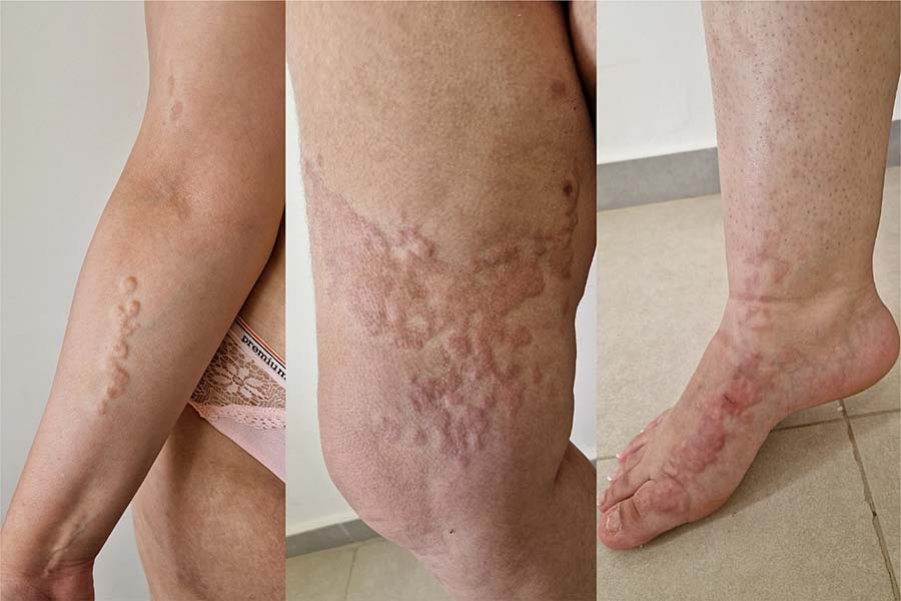
Linear arrangement of flesh-colored papulonodules following Blaschko’s lines on the right limb.

The present case concerns a 25-year-old female patient who presented with skin lesions that had been developing since age 14. A dermatological examination revealed multiple firm, flesh-colored papulonodular lesions that were slightly xanthelasma-like, with some coalescing into plaques. The lesions were strictly confined to the right side of the body, affecting both the trunk and the limbs, and were distributed in a linear pattern along Blaschko's lines (Figs 1 and 2). No lesions were observed on the contralateral side. The lesions were asymptomatic, with no pain or itching.

**Figure 3:**
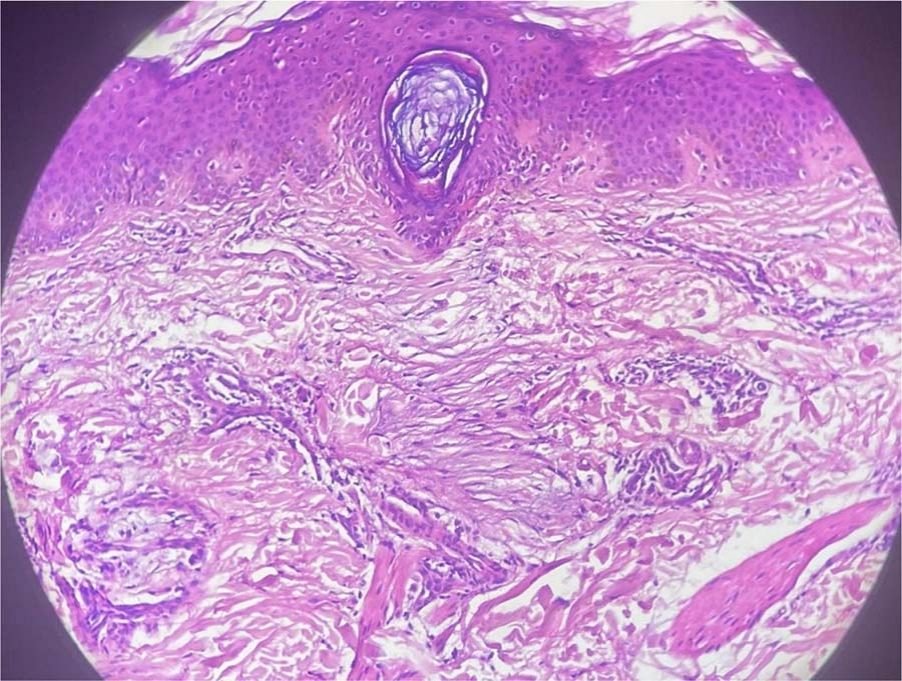
Histopathological section showing abundant mucin deposition within the dermis beneath a normal epidermis, associated with a mild perivascular lymphocytic infiltrate (H&E stain, ×200).

**Figure 4:**
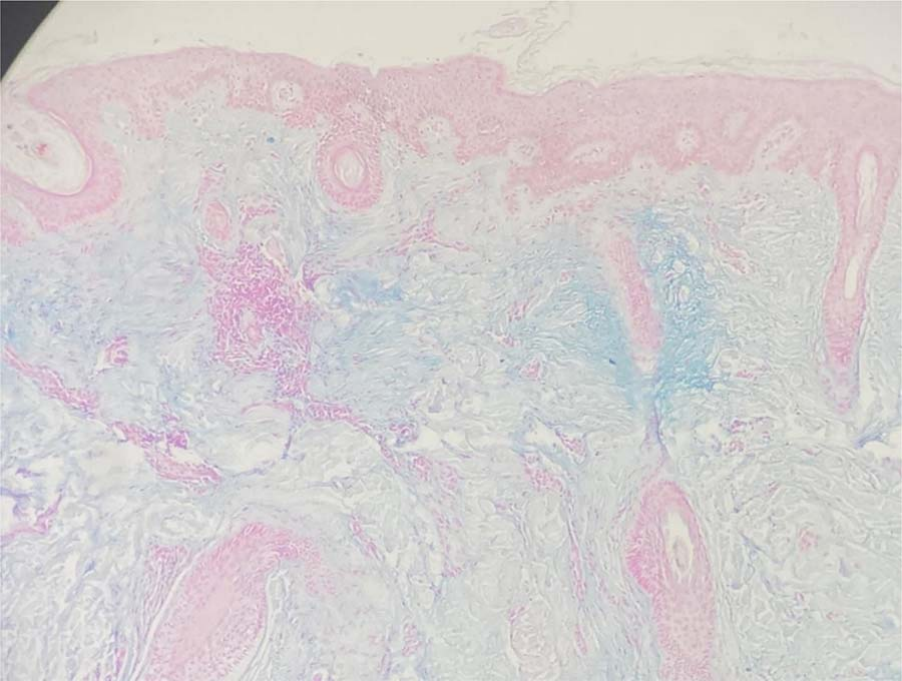
Alcian blue staining demonstrating abundant dermal mucin deposition, confirming the diagnosis of primary cutaneous mucinosis (×200).

A general physical examination revealed no abnormalities. Laboratory tests, including thyroid function, autoimmune screening, and hematological workup, were normal. A skin biopsy revealed abundant mucin deposition in the dermis under a normal epidermis, accompanied by a mild perivascular lymphocytic infiltrate (Fig. 3). Alcian Blue staining confirmed the mucinous nature of the deposits (Fig. 4). There was no evidence of fibroblast proliferation, systemic disease, or generalized mucinosis.

The patient was monitored clinically. During follow-up, no progression of the lesions beyond the right hemibody or systemic involvement was observed.

## Discussion

Primary cutaneous mucinoses are a rare and heterogeneous group of dermatoses characterized by mucin deposition in the dermis without systemic disease or thyroid dysfunction. Among them, localized forms such as lichen myxedematosus typically present as small papules, nodules, or plaques, most often symmetrically distributed. In contrast, generalized forms, like scleromyxedema, are associated with paraproteinemia and systemic manifestations that may threaten prognosis [1, 2].

The case we present stands out because of its strictly unilateral blaschkoid distribution, which, to our knowledge, has not been previously reported in the literature. Blaschko’s lines are classically associated with genetic mosaicism, representing the embryonic migration patterns of keratinocytes and fibroblasts [3]. While such patterns are common in congenital genodermatoses (e.g. epidermal nevi, incontinentia pigmenti) and some acquired inflammatory dermatoses, they remain exceptional in connective tissue disorders such as cutaneous mucinoses. This raises the intriguing possibility that localized primary mucinoses may, at least in some cases, represent a form of cutaneous mosaicism due to postzygotic mutations that alter fibroblast function.

From a histopathological perspective, our findings were consistent with a localized mucinosis: abundant dermal mucin deposition highlighted by Alcian Blue, without evidence of fibroblast proliferation, sclerosis, or inflammatory destruction. This allowed us to exclude scleromyxedema, scleredema, thyroid dermopathy, or secondary mucinosis associated with autoimmune or hematological disease [4]. The absence of systemic symptoms, paraproteinemia, and thyroid dysfunction further supported the diagnosis of a localized, benign entity.

The pathogenesis of mucinoses remains incompletely understood. Current hypotheses suggest abnormal fibroblast stimulation leading to increased glycosaminoglycan production, potentially triggered by cytokines such as TGF-β, IL-1, or TNF-α [5]. In systemic forms, monoclonal gammopathy has been implicated in driving fibroblast activation [2]. In our patient, however, no such systemic factor was identified, making a localized intrinsic fibroblast abnormality more plausible. The blaschkoid distribution reinforces the idea that this abnormality could result from somatic mosaicism, where a subset of fibroblasts carries a mutation leading to focal overproduction of mucin.

From a clinical standpoint, the recognition of such atypical presentations is crucial. Blaschkoid dermatoses often lead clinicians to suspect genodermatoses or inflammatory linear dermatoses; however, this case highlights the importance of including mucinoses in the differential diagnosis. Furthermore, the strictly unilateral distribution reassures about the benign course, distinguishing it from generalized mucinoses that require close systemic monitoring.

## Conclusion

This case illustrates an exceptional presentation of primary cutaneous mucinosis, limited to one hemibody and distributed along Blaschko’s lines. Such a pattern, not previously described, raises the hypothesis of genetic mosaicism as a potential pathogenic mechanism in some localized mucinoses. Recognition of this rare distribution is essential to avoid misdiagnosis and unnecessary investigations, while emphasizing the benign course in the absence of systemic involvement. Future studies, including molecular genetic analyses, are needed to better elucidate the mechanisms underlying such unusual presentations and to expand the clinical spectrum of cutaneous mucinoses.
